# Development and validation of prognostic nomogram for patients with metastatic gastric adenocarcinoma based on the SEER database

**DOI:** 10.1097/MD.0000000000033019

**Published:** 2023-03-03

**Authors:** Xianming Liu, Yanyan Ren, Fayan Wang, Yuqing Bu, Lili Peng, Jinlong Liang, Xiyun Kang, Hongzhen Zhang

**Affiliations:** a Graduate School of Hebei North University, Zhangjiakou, China; b Department of Oncology, Hebei General Hospital, Shijiazhuang, China.

**Keywords:** gastric adenocarcinoma, metastatic, nomogram, overall survival, SEER

## Abstract

The aim of this study was to investigate the prognostic factors affecting overall survival in patients with metastatic gastric adenocarcinoma and to establish a nomogram prediction model for comprehensive clinical application. Data from 2370 patients with metastatic gastric adenocarcinoma between 2010 and 2017 were retrieved from the surveillance, epidemiology, and end results database. They were randomly divided into a training set (70%) and a validation set (30%), univariate and multivariate Cox proportional hazards regressions were used to screen important variables that may affect overall survival and to establish the nomogram. The nomogram model was evaluated using a receiver operating characteristic curve, calibration plot, and decision curve analysis. Internal validation was performed to test the accuracy and validity of the nomogram. Univariate and multivariate Cox regression analyses revealed that, age, primary site, grade, and American joint committee on cancer. T, bone metastasis, liver metastasis, lung metastasis, tumor Size, and chemotherapy were identified as independent prognostic factors for overall survival and were included in the prognostic model to construct a nomogram. The prognostic nomogram showed good overall survival risk stratification ability for the area under the curve, calibration plots, and decision curve analysis in both the training and validation sets. Kaplan–Meier curves further showed that patients in the low-risk group had better overall survival. This study synthesizes the clinical, pathological, therapeutic characteristics of patients with metastatic gastric adenocarcinoma, establishes a clinically effective prognostic model, and that can help clinicians to better evaluate the patient’s condition and provide accurate treatment.

## 1. Introduction

Gastric cancer remains an important cancer worldwide, and according to global cancer statistics in 2020, there have been more than 1 million new cases of gastric cancer and nearly 769,000 deaths. Gastric cancer has become the cancer with the fifth incidence and fourth mortality. Among these, East Asia had the highest incidence of gastric cancer.^[1]^ In China, the incidence and mortality of gastric cancer ranks third, seriously threatening human life.^[2]^ Helicobacter pylori has been confirmed to be closely related to the occurrence and development of gastric cancer, and can be found in almost all patients with confirmed gastric cancer.^[3]^ Risk factors for gastric cancer include smoking, alcohol consumption, and eating pickled foods.^[4]^ A recent meta-analysis found a positive association between consumption of red and processed meat and gastric cancer, with hazard ratios of 1.41 and 1.57, respectively.^[5]^ Thus, a high intake of processed meat, roasted meat, or roasted fish as well as a low intake of fresh fruits can also increase the risk of gastric cancer.^[6]^ Simultaneously, the prognosis and survival of patients with gastric cancer deserve attention.

Although gastric cancer screening has become more common in recent years with advances in endoscopic screening techniques and increased risk awareness, most patients still present with distant metastasis. Among them, 16.92%, 5.92%, 5.08%, and 0.79% developed liver, lung, bone and brain metastases, respectively.^[7]^ Currently, there is no curative treatment for advanced metastatic gastric adenocarcinoma. Fortunately, chemotherapy and immunotherapy have been approved and have achieved good therapeutic results in the treatment of advanced metastatic gastric adenocarcinoma.^[8]^ Despite these results, most patients with metastatic gastric adenocarcinoma still have pessimistic survival, with a median survival of only about 10.6 to 17.4 months.^[9]^ With the development of new treatment methods and changes in treatment modalities, patients with metastatic gastric adenocarcinoma will achieve better survival.

Currently, the prognosis of gastric cancer is based on the TNM stage system issued by the union for international cancer control and the American joint committee on cancer (AJCC).^[10]^ Because TNM stage only includes the pathological features of the tumor itself, clinical features such as age, sex, marital status, and treatment that can also determine the prognosis are not included.^[11,12]^ Therefore, we could not accurately evaluate the prognosis of patients with gastric cancer, especially metastatic gastric adenocarcinomas. Accordingly, there is an urgent need to develop a new prognostic prediction model to accurately predict and obtain the possibility of individual survival outcomes, to carry out precision treatment. A nomogram is a statistical tool that visualizes findings and can encompass both clinical and pathological variables, with both statistical and visual advantages. In view of the need for large sample studies, this study was based on the surveillance, epidemiology, and end results (SEER) database to analyze demographic characteristics, clinical features, and pathological features to predict patient survival. In addition, this study developed and validated accurate and individualized prognostic nomograms for overall survival (OS) in patients with metastatic gastric adenocarcinoma and assessed the appropriate treatment modalities.

## 2. Methods

### 2.1. Patient

**Figure 1.**
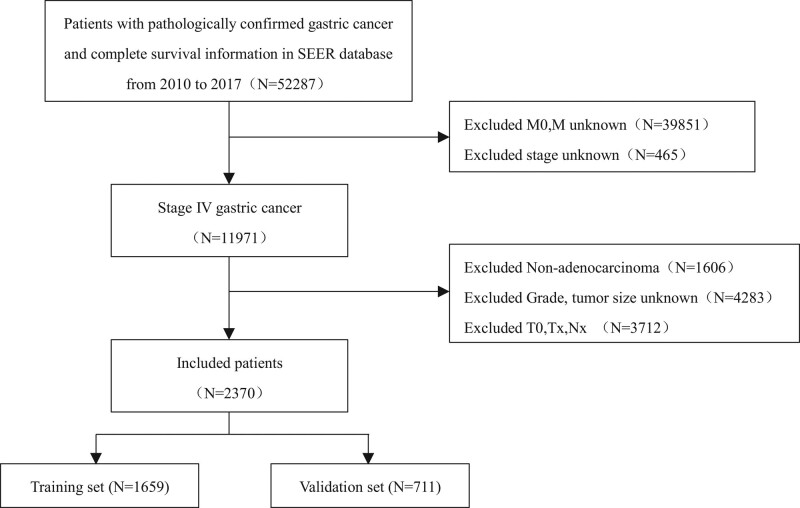
The inclusion criteria flowchart of recruited patients in SEER database. SEER = surveillance, epidemiology, and end results database.

Patients in the development and validation cohorts in this study were recruited from the SEER database supported by the National Cancer Institute, which collects information on the clinical characteristics and survival of approximately 1-third of cancer patients in the United States. SEER Stat software (version 8.4.0) (https://seer.cancer.gov/) was used to extract patient clinical information. We collected data on patients with metastatic gastric adenocarcinoma using ICD-0-3/ world health organization 2008 histology codes 8140/3 (adenocarcinoma, NOS), 8144/3 (adenocarcinoma, intestinal type), 8210/3 (adenocarcinoma in situ within adenomatous polyp), 8211/3 (tubular adenocarcinoma), 8255/3 (adenocarcinoma, mixed subtype), 8260/3 (papillary adenocarcinoma), 8261/3 (adenocarcinoma within villous adenoma), 8263/3 (adenocarcinoma within tubular villous adenoma), 8323/3 (mixed cell adenocarcinoma), 8480/3 (mucinous adenocarcinoma), 8481/3 (secretory mucinous adenocarcinoma), and 8490/3 (signet ring cell carcinoma). Since the SEER database has collected distant metastasis data since 2010, patients with gastric adenocarcinoma from 2010 to 2017 were searched according to the AJCC 7th edition TNM staging system. The exclusion criteria for our study were as follows: Unknown metastatic site; Incomplete clinical, pathological, and treatment information required; Multiple primary tumor lesions; and survival time of < 3 months. All patients with metastatic gastric adenocarcinoma included in the study were randomly matched to the training and validation sets at a 7:3 ratio (Fig. 1). Because the data used in this study were retrieved from the SEER database using publicly available methods, ethical approval or claims were not required.

### 2.2. Data collection and endpoint definition

**Figure 2.**
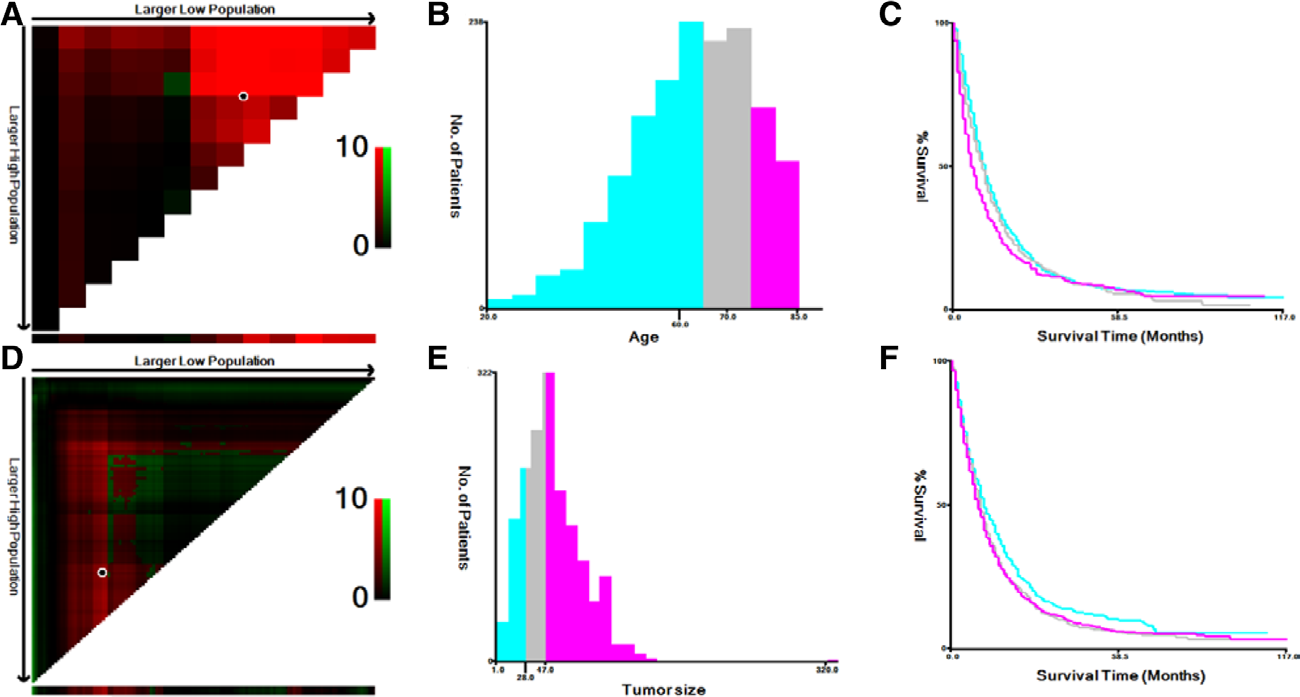
The X-tile analysis of best-cutoff points of age and tumor size variables. (A) X-tile plot of training sets in age; (B) the cutoff point was highlighted using a histogram of the entire cohort; (C) the distinct prognosis determined by the cutoff point was shown using a Kaplan–Meier plot (low subset = blue, middle subset = gray, high subset = magenta); (D) X-tile plot of training sets in tumor size; (E) the cutoff point was highlighted using a histogram; (F) Kaplan–Meier plot of prognosis determined by the cutoff point (low subset = blue, middle subset = gray, high subset = magenta).

The following data were collected for all eligible patients, age, sex, marital status, primary tumor site, histological type, differentiation, TNM stage, clinical stage, tumor size, bone metastasis, brain metastasis, liver metastasis, lung metastasis, and treatment information and survival outcome. In this study, 2 variables, age and tumor size, were grouped and visualized using the X-tile software to determine the optimal cutoff value (Fig. 2). According to the SEER database, the primary sites of the tumors included C16.0 (cardia), C16.1 (fundus), C16.2 (corpus), C16.3 (antrum), C16.4 (pylorus), C16.5 (lesser curvature), C16.6 (greater curvature), C16.8 (partially overlapping gastric lesion), and C16.9 (stomach, NOS). The patients marital status was divided into 3 groups: married, unmarried and unknown. Histological grades were classified according to the world health organization criteria as well-differentiated (grade I), moderately differentiated (grade II), poorly differentiated (grade III), and undifferentiated (grade IV). The primary endpoint was OS. OS was defined as the time from the date of the primary diagnosis of metastatic gastric cancer to the date of death from any cause.

### 2.3. Statistical analysis

Categorical variables are presented as frequencies and rates and were tested using the chi-square test. Univariate and multivariate Cox proportional hazards models were used to select independent prognostic factors for OS, and nomogram models were constructed to predict the OS at 6, 12, and 24 months. The prognostic models were internally validated using a cohort of 711 patients from the SEER database. Parameters for Cox proportional hazards regression analysis included hazard ratios and the corresponding 95% confidence intervals. The receiver operating characteristic curve (ROC) was used to hire the discrimination power of the current prediction model, and a larger area under the curve (AUC) ROC indicated a better model. Calibration plots were used for visual comparison of the nomogram-predicted prognosis with the actual prognosis. Decision curve analysis (DCA) was performed to assess the clinical validity of the predictive model. Statistical significance was set at 2-sided *P* < .05. All statistical analyses were performed using the R software (version 4.2.1) (http://www.r-projec.org/).

## 3. Results

### 3.1. Patient characteristics

We extracted data from 2370 patients from the SEER database and randomly divided them into a training set (1659 patients) and validation set (711 patients) in a 7:3ratio. The median follow-up durations were 13 months and 10 months for the training and validation sets, respectively. Table 1 presents the demographic and clinical characteristics of patients with metastatic gastric cancer in the training and validation sets.

**Table 1:** Patients’ demographics and clinicopathological characteristics.

Variables	All patients (n = 2370)	Training set (n = 1659)	Validation set (n = 711)	*P* value
Sex (%)				
Male	1597 (67.4)	1124 (67.8)	473 (66.5)	.592
Female	773 (32.6)	535 (32.2)	238 (33.5)	
Age (%)				
<=60	1201 (50.7)	845 (50.9)	356 (50.1)	.078
61–70	627 (26.5)	454 (27.4)	173(24.3)	
>=71	542 (22.8)	360 (21.7)	182(25.6)	
Married status (%)				
Unmarried	993 (41.8)	708 (42.7)	285 (40.2)	.466
Married	1281 (54.1)	883 (53.2)	398 (56.0)	
Unknown	96 (4.1)	68 (4.1)	28 (3.9)	
Primary site (%)				
Antrum	447 (18.9)	311 (18.7)	136 (19.1)	.415
Body	211 (8.9)	153 (9.2)	58 (8.2)	
Cardia	883 (37.3)	626 (37.7)	257 (36.1)	
Fundus	74 (3.1)	52 (3.1)	22 (3.1)	
Greater	116 (4.9)	76 (4.6)	40 (5.6)	
Lesser	182 (7.7)	136 (8.2)	46 (6.5)	
Overlapping	226 (9.5)	146 (8.8)	80 (11.3)	
Pylorus	57 (2.4)	42 (2.5)	15 (2.1)	
Stomach, NOS	174 (7.3)	117 (7.1)	57 (8.0)	
Grade (%)				
I	50 (2.1)	34 (2.0)	16 (2.3)	.473
II	588 (24.8)	415 (25.1)	173 (24.3)	
III	1676 (70.7)	1176 (70.9)	500 (70.3)	
IV	56 (2.4)	34 (2.0)	22 (3.1)	
AJCC.T (%)				
T1	481 (20.3)	335 (20.2)	146 (20.5)	.846
T2	150 (6.3)	107 (6.4)	43 (6.0)	
T3	812 (34.3)	576 (34.7)	236 (33.2)	
T4	927 (39.1)	641 (38.7)	286 (40.2)	
AJCC.N (%)				
N0	602 (25.4)	422 (25.4)	180 (25.3)	.976
N1	902 (38.1)	633 (38.1)	269 (37.8)	
N2	366 (15.4)	258 (15.6)	108 (15.2)	
N3	500 (21.1)	346 (20.9)	154 (21.7)	
Bone metastasis (%)				
No	2091 (88.2)	1465 (88.3)	626 (88.0)	.429
Yes	238 (10.1)	162 (9.8)	76 (10.7)	
Unknown	41 (1.7)	32 (1.9)	9 (1.3)	
Brain metastasis (%)				
No	2297 (96.9)	1602 (96.6)	695 (97.7)	.308
Yes	33 (1.4)	26 (1.6)	7 (1.0)	
Unknown	40 (1.7)	31 (1.8)	9 (1.3)	
Liver metastasis (%)				
No	1404 (59.2)	994 (59.9)	410 (57.7)	.089
Yes	934 (39.4)	638 (38.5)	296 (41.6)	
Unknown	32 (1.4)	27 (1.6)	5 (0.7)	
Lung metastasis (%)				
No	2060 (86.9)	1436 (86.6)	624 (87.8)	.102
Yes	265 (11.2)	185 (11.1)	80 (11.2)	
Unknown	45 (1.9)	38 (2.3)	7 (1.0)	
Tumor size (cm, %)				
<=2.8	376 (15.9)	273 (16.5)	103 (14.5)	.432
2.9–4.7	658 (27.7)	462 (27.8)	196 (27.6)	
>=4.8	1336 (56.4)	924 (55.7)	412 (57.9)	
Radiotherapy (%)				
No	1751 (73.9)	1214 (72.2)	537 (75.5)	.133
Yes	619 (26.1)	445 (26.8)	174 (24.5)	
Chemotherapy (%)				
No	528 (22.3)	359 (21.6)	169 (23.8)	.277
Yes	1842 (77.7)	1300 (78.4)	542 (76.2)	

AJCC = American joint committee on cancer.

The characteristics of patients in the training set were similar to those in the validation set. Among them, 67.4% were males, 32.6% were females, and 50.7% were < = 60 years of age. 61 to 70 years old accounted for 26.5%, > = 71 years old accounted for 22.8%; bone metastasis accounted for 10.1%, brain metastasis accounted for 1.4%, liver metastasis accounted for 39.4%, lung metastasis accounted for 11.2%. Patients with tumor size < = 2.8 cm, 2.9 to 4.7 cm and > = 4.8 cm accounted for 15.9%,27.7%, and 56.4% of the included patients, respectively. With regard to treatment modality, radiotherapy was performed in 26.1% of patients and chemotherapy in 77.7% of patients. No significant differences were found between the training and validation sets for any variable included in this study.

### 3.2. Determining the predictors

Univariate analysis of OS in this study is shown in Table 2. The results showed that age, primary site, differentiation, AJCC.T stage, bone metastasis, brain metastasis, liver metastasis, lung metastasis, tumor size and chemotherapy were important prognostic factors. Variables with a *P* value of < .05 were included in the multivariate analysis, and the results showed that age, primary site, differentiation, AJCC.T, bone metastasis, liver metastasis, lung metastasis, tumor size, and chemotherapy were independent prognostic factors for OS in patients with metastatic gastric adenocarcinoma. As shown in Table 3.

**Table 2:** Univariate analysis of patients with metastatic gastric adenocarcinoma.

Variables	HR (95% CI)	*P* value
Sex		
Female vs male	1.041 (0.935–1.159)	.467
Age (yr)		
<=60 vs 61–70	1.083 (0.961–1.220)	.190
<=60 vs >=71	1.271 (1.117–1.445)	< .001
Marital status		
Married vs unmarried	0.986 (0.891–1.095)	.812
Married vs unknown	1.013 (0.784–1.309)	.921
Primary site		
Antrum vs body	0.992 (0.809–1.218)	.942
Antrum vs cardia	1.125 (0.975–1.297)	.107
Antrum vs fundus	1.119 (0.827–1.517)	.465
Antrum vs greater	1.218 (0.934–1.587)	.145
Antrum vs lesser	1.072 (0.868–1.324)	.518
Antrum vs overlapping	1.264 (1.029–1.553)	.025
Antrum vs pylorus	1.080 (0.769–1.516)	.655
Antrum vs stomach, NOS	1.116 (0.892–1.397)	.337
Grade		
I vs II	1.102 (0.755–1.608)	.615
I vs III	1.395 (1.965–2.018)	.001
I vs IV	1.112 (0.664–1.861)	.688
AJCC.T		
T1 vs T2	0.580 (0.459–0.733)	< .001
T1 vs T3	0.809 (0.704–0.931)	.003
T1 vs T4	0.975 (0.851–1.118)	.719
AJCC.N		
N0 vs N1	1.006 (0.885-1.143)	.931
N0 vs N2	0.901 (0.766–1.060)	.210
N0 vs N3	1.003 (0.865–1.163)	.967
Bone metastasis		
No vs yes	1.847 (1.561–2.185)	< .001
No vs unknown	1.675 (1.172–2.394)	.005
Brain metastasis		
No vs yes	1.564 (1.053–2.324)	.026
No vs unknown	1.743 (1.213–2.504)	.002
Liver metastasis		
No vs yes	1.215 (1.096–1.347)	< .001
No vs Unknown	1.526 (1.033–2.255)	.033
Lung metastasis		
No vs yes	1.355 (1.157–1.588)	< .001
No vs Unknown	1.814 (1.313–2.508)	< .001
Tumor size (cm)		
<=2.8 vs 2.9–4.7	1.178 (1.007–1.378)	.040
<=2.8 vs >=4.8	1.195 (1.036–1.377)	.014
Radiotherapy		
No vs yes	0.994 (0.887–1.114)	.919
Chemotherapy		
No vs yes	0.529 (0.467–0.597)	< .001

AJCC = American joint committee on cancer, CI = confidence interval, HR = hazard ratio.

**Table 3:** Multivariate analysis of patients with metastatic gastric adenocarcinoma.

Variables	HR (95% CI)	*P* value
Age (yr)		
<=60 vs 61–70	1.066 (0.942–1.205)	.310
<=60 vs >=71	1.186 (1.033–1.363)	.016
Primary site		
Antrum vs body	1.069 (0.868–1.316)	.530
Antrum vs cardia	1.251 (1.071–1.462)	.004
Antrum vs fundus	1.023 (0.752–1.392)	.885
Antrum vs greater	1.102 (0.841–1.443)	.481
Antrum vs lesser	1.128 (0.911–1.397)	.269
Antrum vs overlapping	1.383 (1.123–1.705)	.002
Antrum vs pylorus	0.960 (0.679–1.358)	.818
Antrum vs stomach, NOS	1.048 (0.836–1.315)	.685
Grade		
I vs II	1.254(0.855-1.837)	.247
I vs III	1.697(1.167-2.469)	.005
I vs IV	1.165(0.688-1.972)	.570
AJCC.T		
T1 vs T2	0.601 (0.473–0.764)	< .001
T1 vs T3	0.841 (0.729–0.970)	.018
T1 vs T4	1.034 (0.889–1.203)	.663
Bone metastasis		
No vs yes	1.819 (1.527–2.166)	< .001
No vs unknown	0.673 (0.252–1.799)	.430
Liver metastasis		
No vs yes	1.274 (1.142–1.421	< .001
No vs unknown	1.209 (0.706–2.072)	.488
Lung metastasis		
No vs yes	1.364 (1.158–1.607)	< .001
No vs unknown	1.277 (0.845–2.026)	.299
Tumor size (cm)		
<=2.8 vs 2.9–4.7	1.206 (1.028–1.415)	.021
<=2.8 vs >=4.8	1.186 (1.027–1.376)	.020
Chemotherapy		
No vs yes	0.497 (0.435–0.569)	< .001

AJCC = American joint committee on cancer, CI = confidence interval, HR = hazard ratio.

### 3.3. Developing and validating the nomogram

**Figure 3.**
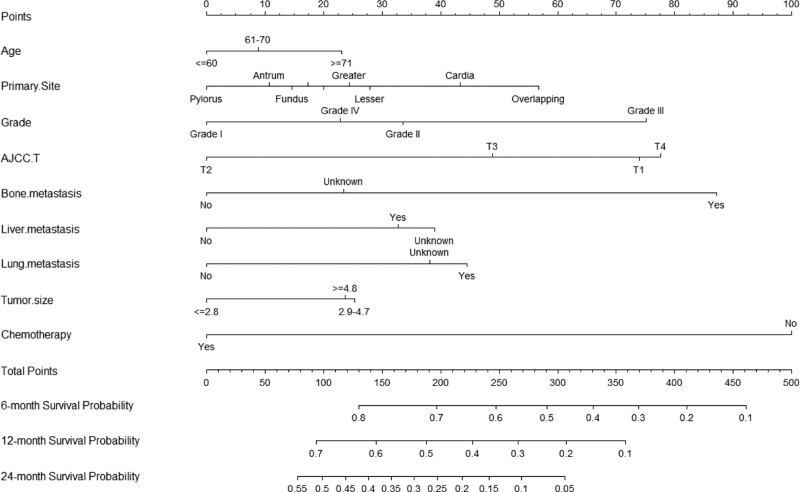
Nomogram for predicting 6-, 12-, and 24-month OS of patients with metastatic gastric adenocarcinoma. For each patient, 9 lines are drawn up to determine the points received from the predictors in the line plot. The sum of these points is on the Total Points axis. In addition, 3 lines are drawn down to determine the possibility of 6, 12, and 24 months. OS = overall survival.

**Figure 4.**
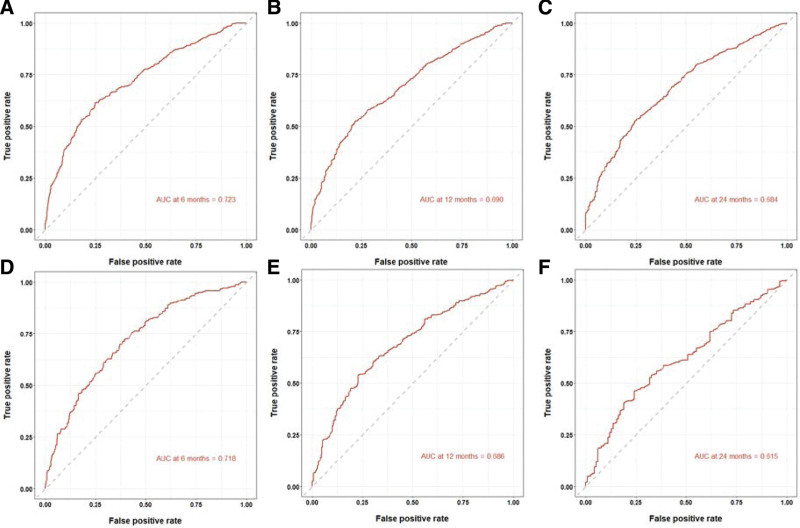
ROC curves of the nomogram in the prediction of prognosis. 6-month OS (A) and 12-month OS (B) and 24-month OS (C) in the training set; 6-month OS (D) and 12-month OS (E) and 24-month OS (F) in validation set. ROC = receiver operating characteristic curve, AUC = areas under the curve, OS = overall survival.

**Figure 5.**
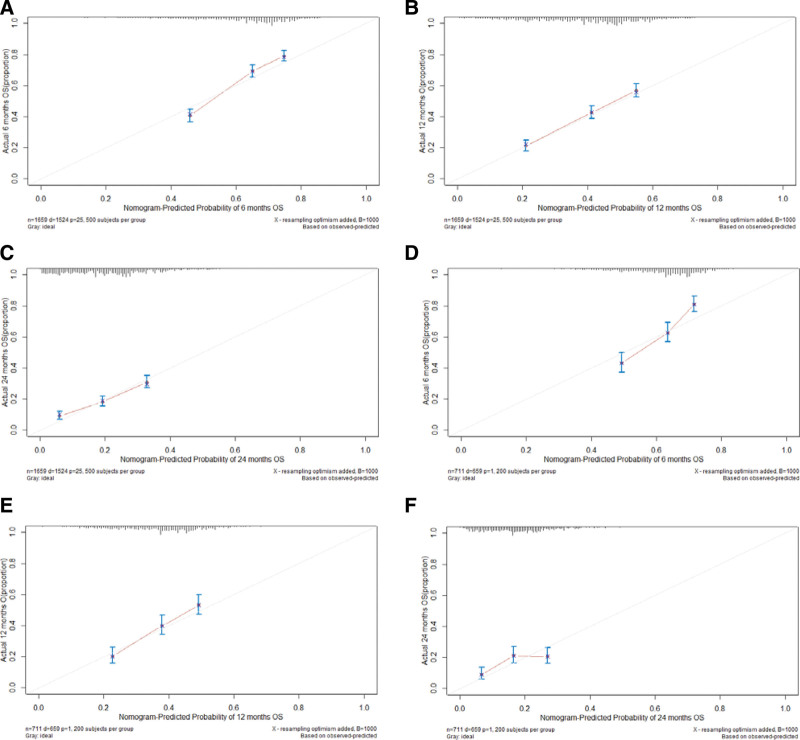
Calibration plots of OS nomogram model. 6-month calibration plot of OS using training set (A); 12-month calibration plot of OS using training set (B); 24-month calibration plot of OS using training set (C);6-month calibration plot of OS using validation set (D);12-month calibration plot of OS using validation set (E);24-month calibration plot of OS using validation set (F). OS = overall survival.

**Figure 6.**
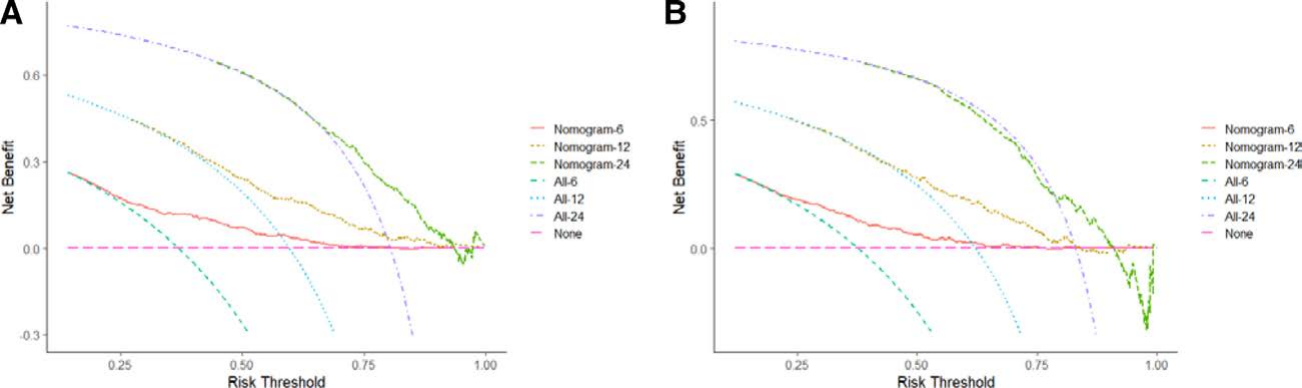
Decision curve analysis for the nomogram in the prediction of prognosis of patients with metastatic gastric adenocarcinoma. The Decision curve analysis of the training set (A) and validation set (B). The x-axis shows the threshold probabilities, and the y-axis measures the net benefit calculated by adding the true positives and subtracting the false positives.

**Figure 7.**
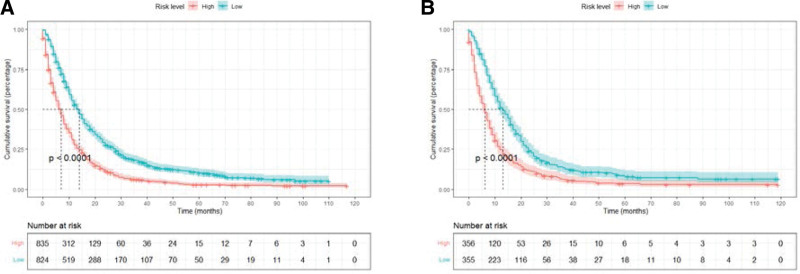
Kaplan–Meier curves of overall survival (OS) for patients in different risk levels. The survival of the low- and high-risk groups in the training set (A), validation set(B).

A nomogram was constructed based on independent prognostic factors derived from multivariate Cox regression analysis to predict the OS at 6, 12, and 24 months in patients with metastatic gastric adenocarcinoma. As shown in Figure 3. Nine lines were drawn to identify the points of the predictor variables in the model, and the corresponding scores were assigned to each predictor variable. The sum of these scores could lie on the total number of points line. Finally, 6-month, 12-month, and 24-month survival rates can be predicted according to the line of probability of survival. The same 711 patients from the SEER database constituted the validation set used to validate this predictive model. The AUCs in the training set were 0.723, 0.690 and 0.684 at 6-month, 12-month, and 24-month, respectively (Fig. 4A, B, C), and 0.718, 0.686, and 0.615 in the validation set (Fig. 4D–F). The AUC value suggests that this predictive model is highly discriminative. Calibration plots were used to validate the ability of the model to predict 6, 12, and 24 months in patients with metastatic gastric adenocarcinoma. In both the training (Fig. 5A–C) and validation (Fig. 5D–F) sets, the calibration plots were close to the diagonal line, indicating that nomogram predictions were perfectly correlated with the observed results, confirming the reliability of the nomogram. At the same time, DCA plots showed high clinical validity of our prediction model (Fig. 6). Based on the nomogram, we calculated the total and median survival scores of patients with metastatic gastric cancer in the training and validation sets, respectively, and divided all patients into 2 groups according to the median: those with a total score greater than or equal to the median were in the high-risk group and those < the median were in the low-risk group. Survival analysis using Kaplan–Meier curves showed that the low-risk group had significantly better survival outcomes both in the training set (*P* < .0001) and in the validation set (*P* < .0001) than the high-risk group. show in Figure 7.

## 4. Discussion

Adenocarcinoma remains the most common pathological type of gastric malignancy, and metastatic gastric adenocarcinoma remains a serious threat to human health as a heterogeneous disease.^[13]^ In clinical practice, there is a need to accurately predict the prognosis of patients with metastatic gastric adenocarcinoma, thereby improving their survival rate. In addition, the high mortality rate of metastatic gastric adenocarcinoma seriously affects the choice of individualized and precise treatment for patients.^[14]^ Therefore, there is an urgent need to develop a tool that comprehensively considers multiple prognostic factors to accurately predict the survival time and survival probability of patients with metastatic gastric adenocarcinoma. However, some studies have developed nomograms for the treatment of metastatic gastric cancer. For example, Feng, Y^[15]^ and Zhu, Y^[16]^ developed nomograms for metastatic gastric cancer; however, they focused more on early mortality in stage IV gastric cancer. To our knowledge, this study is the first to explore the overall survival of patients with metastatic gastric adenocarcinoma.

As a visualization of multivariate prognostic models, nomograms integrate multiple prognostic factors and can be used to accurately assess the probability of survival of patients at a specific time and to predict the long-term survival of cancer patients.^[17–19]^ Survival prediction in patients with metastatic gastric adenocarcinoma, based on nomograms, was performed in this study. First, univariate and multivariate Cox regression analyses were used to assess the independent prognostic factors for OS. Multivariate analysis showed that age, primary site, grade, AJCC T, bone metastasis, liver metastasis, lung metastasis, tumor size, and chemotherapy were independent prognostic factors for OS in patients with metastatic gastric adenocarcinoma. Accordingly, we established prognostic nomograms for 6-month, 12-month, and 24-month OS in these patients. A key point in predicting a prognostic model is that it can distinguish between high-risk (shorter survival time) and low-risk (longer survival time) populations based on certain factors.^[20]^ The ROC curve, calibration curve, and DCA curve were used to verify the clinical utility and prediction performance of the nomogram, all of which showed that the nomogram was highly superior, and the prediction results were in good agreement with the actual observed results. It should be pointed out that the clinical validity of the DCA curve at 12 months was relatively poor in both the training and validation sets. This phenomenon may be due to the high degree of malignancy and poor behavioral characteristics of metastatic gastric adenocarcinoma, resulting in an overall survival of < 12 months in a significant number of patients.

Previous studies have shown that age is a risk factor affecting the prognosis of gastric cancer, the risk ratio of elderly patients is significantly increased, and the survival time is significantly shortened compared with young patients.^[11,15]^ The findings of this study are identical to those described above. The reasons for the shortened survival time of elderly patients may be as follows. First, with an increase in age, the function of the immune system gradually decreases, and at the same time, elderly patients are more likely to have complications.^[21]^ Second, because younger patients generally have better physical conditions, they are more willing to accept and more easily tolerate multiple treatments including surgical treatment and, chemoradiotherapy.^[22,23]^

The primary tumor site has been shown to be one of the factors affecting the survival outcome of patients.^[24]^ The cardia, fundus, other parts close to the esophagus are called the proximal stomach, and whereas the pylorus and other parts connected to the duodenum are called the distal stomach. Talamonti et al^[25]^ found that the median OS was reduced by half in patients with proximal primary tumors compared to those with distal tumors. This finding is consistent with the results of the present study. The reason for this phenomenon may be that tumors originating in the proximal stomach, such as gastric cardia cancer, have more aggressive biological behavior and are more likely to develop lymph node metastasis.^[26]^

Tumor size is another factor that has been shown to influence the survival outcome of patients, and we usually refer to the maximum diameter of the tumor that can be measured as its size.^[27]^ This indicator is often used to assess whether a tumor is respectable and which resection method is used, thus further affecting the individualized treatment of patients. The tumor size of the vast majority of solid tumors, such as lung cancer and breast cancer, is included in their TNM stage system to assess prognosis; the larger the tumor, the worse the prognosis.^[28,29]^ This finding is consistent with the results of the present study. However, the *T* stage of gastric cancer only includes the depth of tumor invasion, and if the tumor size is used together with the depth of tumor invasion to assess prognosis, it will lead to more precise treatment for patients.

As the tumor burden increases, the tumor develops distant metastases. Liver metastasis is the most common site of distant metastasis in gastric cancer, and the prognosis of this type of patient is poor, with a median OS of only 2 to 3 months.^[30]^ Tumor cells can also metastasize to the bones and lungs via blood circulation. In contrast to liver metastases, lung metastases and bone metastases, although rare, have an unfavorable prognosis, with a median OS of only 3 and 4 months, and respectively.^[7]^ In addition, we found that the worse the degree of differentiation, the greater the likelihood of liver, lung, and bone metastasis. Our study yielded similar results.

This study has several limitations. First, the SEER database does not contain specific information about chemotherapy, radiotherapy, etc, such as the dose of chemotherapy drugs and target volumes, which are also important for the prognosis of patients with metastatic gastric adenocarcinoma. Second, missing data could have led to selection bias. Third, we did not perform an external validation to further evaluate the nomogram. Next, this can be verified by including a large number of patients from different regions to prove that the prediction models have better applicability. Our study also had certain advantages. First, to our knowledge, this study is the first to use a nomogram model to predict overall survival in patients with metastatic gastric adenocarcinoma. Second, this study developed a nomogram based on a large population and conducted internal validation, making the model relatively reliable and helping clinicians provide individualized treatment for patients with metastatic gastric adenocarcinoma.

## Acknowledgment

All authors would like to thank SEER for the open access to the database.

## Author contributions

**Conceptualization:** Xianming Liu, Hongzhen Zhang.

**Data curation:** Yanyan Ren, Fayan Wang.

**Formal analysis:** Jinlong Liang, Xiyun Kang.

**Methodology:** Yanyan Ren.

**Software:** Yuqing Bu, Lili Peng.

**Writing – original draft:** Xianming Liu.

**Writing – review & editing:** Hongzhen Zhang.
